# Supplementary material for: Modeling glioblastoma heterogeneity as a dynamic network of cell states
Source: Mol Syst Biol. 2021 Sep 16;17(9):e10105. doi: 10.15252/msb.202010105 (PMC8444284; doi:10.15252/msb.202010105)
Supplement: Supplementary file 5 — Source Data for Figure 3 [file MSB-17-e10105-s001.zip › Figure3A_sourcedata/GSEA_3065/hallmarks_state5.GseaPreranked.1623416302988/HALLMARK_KRAS_SIGNALING_DN.html]

Details for gene set HALLMARK\_KRAS\_SIGNALING\_DN[GSEA]

|  || Dataset | state5 |
| Phenotype | NoPhenotypeAvailable |
| Upregulated in class | na\_pos |
| GeneSet | HALLMARK\_KRAS\_SIGNALING\_DN |
| Enrichment Score (ES) | 0.22373869 |
| Normalized Enrichment Score (NES) | 0.71726006 |
| Nominal p-value | 0.9140401 |
| FDR q-value | 0.9607933 |
| FWER p-Value | 1.0 |
Table: GSEA Results Summary

  

Fig 1: Enrichment plot: HALLMARK\_KRAS\_SIGNALING\_DN      
 Profile of the Running ES Score & Positions of GeneSet Members on the Rank Ordered List

  

| PROBE | GENE SYMBOL | GENE\_TITLE | RANK IN GENE LIST | RANK METRIC SCORE | RUNNING ES | CORE ENRICHMENT || 1 | IGFBP2 |  |  | 137 | 0.491 | 0.1073 | Yes |
| 2 | FGFR3 |  |  | 567 | 0.293 | 0.1323 | Yes |
| 3 | CELSR2 |  |  | 750 | 0.251 | 0.1745 | Yes |
| 4 | YPEL1 |  |  | 1363 | 0.165 | 0.1473 | Yes |
| 5 | LFNG |  |  | 1442 | 0.158 | 0.1779 | Yes |
| 6 | KMT2D |  |  | 1946 | 0.116 | 0.1506 | Yes |
| 7 | TGFB2 |  |  | 2112 | 0.104 | 0.1582 | Yes |
| 8 | BTG2 |  |  | 2174 | 0.100 | 0.1763 | Yes |
| 9 | GTF3C5 |  |  | 2222 | 0.097 | 0.1952 | Yes |
| 10 | PTPRJ |  |  | 2325 | 0.090 | 0.2063 | Yes |
| 11 | VPS50 |  |  | 2611 | 0.073 | 0.1927 | Yes |
| 12 | SPHK2 |  |  | 2631 | 0.072 | 0.2085 | Yes |
| 13 | DCC |  |  | 2725 | 0.067 | 0.2148 | Yes |
| 14 | CDKAL1 |  |  | 2787 | 0.063 | 0.2237 | Yes |
| 15 | TCF7L1 |  |  | 3012 | 0.051 | 0.2115 | No |
| 16 | SLC38A3 |  |  | 3125 | 0.046 | 0.2103 | No |
| 17 | KCNQ2 |  |  | 3165 | 0.043 | 0.2168 | No |
| 18 | SKIL |  |  | 3291 | 0.037 | 0.2120 | No |
| 19 | MFSD6 |  |  | 3654 | 0.022 | 0.1770 | No |
| 20 | CAMK1D |  |  | 4717 | -0.023 | 0.0641 | No |
| 21 | BARD1 |  |  | 4776 | -0.025 | 0.0638 | No |
| 22 | KLHDC8A |  |  | 5283 | -0.043 | 0.0180 | No |
| 23 | SHOX2 |  |  | 5705 | -0.062 | -0.0135 | No |
| 24 | CCDC106 |  |  | 6197 | -0.085 | -0.0472 | No |
| 25 | EFHD1 |  |  | 6534 | -0.104 | -0.0587 | No |
| 26 | COPZ2 |  |  | 6537 | -0.104 | -0.0329 | No |
| 27 | CHST2 |  |  | 6560 | -0.106 | -0.0091 | No |
| 28 | NUDT11 |  |  | 6758 | -0.118 | -0.0017 | No |
| 29 | PDK2 |  |  | 7392 | -0.180 | -0.0275 | No |
| 30 | SNN |  |  | 7577 | -0.201 | 0.0020 | No |
| 31 | SLC25A23 |  |  | 7679 | -0.214 | 0.0442 | No |
| 32 | GAMT |  |  | 8449 | -0.407 | 0.0598 | No |
Table: GSEA details [plain text format]

  

Fig 2: HALLMARK\_KRAS\_SIGNALING\_DN: Random ES distribution      
 Gene set null distribution of ES for **HALLMARK\_KRAS\_SIGNALING\_DN**
